# A Novel ATM/TP53/p21-Mediated Checkpoint Only Activated by Chronic γ-Irradiation

**DOI:** 10.1371/journal.pone.0104279

**Published:** 2014-08-05

**Authors:** Lili Cao, Hidehiko Kawai, Megumi Sasatani, Daisuke Iizuka, Yuji Masuda, Toshiya Inaba, Keiji Suzuki, Akira Ootsuyama, Toshiyuki Umata, Kenji Kamiya, Fumio Suzuki

**Affiliations:** 1 Department of Experimental Oncology, Research Institute for Radiation Biology and Medicine, Hiroshima University, Hiroshima, Japan; 2 Department of Molecular Radiobiology, Research Institute for Radiation Biology and Medicine, Hiroshima University, Hiroshima, Japan; 3 Department of Genome Dynamics, Research Institute of Environmental Medicine, Nagoya University, Furo-cho, Chikusa-ku, Nagoya, Japan; 4 Department of Molecular Oncology & Leukemia Program Project, Research Institute for Radiation Biology and Medicine, Hiroshima University, Hiroshima, Japan; 5 Department of Radiation Medical Sciences, Atomic Bomb Disease Institute, Nagasaki University, Nagasaki, Japan; 6 Department of Radiation Biology and Health, School of Medicine, University of Occupational and Environmental Health, Kitakyushu, Japan; 7 Radioisotope Research Center, University of Occupational and Environmental Health, Kitakyushu, Japan; ENEA, Italy

## Abstract

Different levels or types of DNA damage activate distinct signaling pathways that elicit various cellular responses, including cell-cycle arrest, DNA repair, senescence, and apoptosis. Whereas a range of DNA-damage responses have been characterized, mechanisms underlying subsequent cell-fate decision remain elusive. Here we exposed cultured cells and mice to different doses and dose rates of γ-irradiation, which revealed cell-type-specific sensitivities to chronic, but not acute, γ-irradiation. Among tested cell types, human fibroblasts were associated with the highest levels of growth inhibition in response to chronic γ-irradiation. In this context, fibroblasts exhibited a reversible G1 cell-cycle arrest or an irreversible senescence-like growth arrest, depending on the irradiation dose rate or the rate of DNA damage. Remarkably, when the same dose of γ-irradiation was delivered chronically or acutely, chronic delivery induced considerably more cellular senescence. A similar effect was observed with primary cells isolated from irradiated mice. We demonstrate a critical role for the ataxia telangiectasia mutated (ATM)/tumor protein p53 (TP53)/p21 pathway in regulating DNA-damage-associated cell fate. Indeed, blocking the ATM/TP53/p21 pathway deregulated DNA damage responses, leading to micronucleus formation in chronically irradiated cells. Together these results provide insights into the mechanisms governing cell-fate determination in response to different rates of DNA damage.

## Introduction

Lesions to genomic DNA, including modified bases, and single- and double-strand breaks, are constantly generated in living cells under physiological and environmental conditions [1]. DNA damage can result from internal or external sources and cause mutations to genomic DNA. These lesions and mutations to genomic DNA affect cell-fate outcomes (e.g., proliferation, cell-cycle arrest, senescence, differentiation, autophagy, transformation, and apoptosis), which are directly linked to human-health impairments, including cancer and aging [2]. The overall rate of spontaneous DNA damage in human cells is estimated to be tens of thousands of events per day, which is approximately equivalent to the rate induced by exposure to sparsely ionizing radiation (1.5–2.0 Gray (Gy)/day) [3], . Under these conditions, individual cells adopt particular cell fates to maintain homeostasis within the living organisms. As cell fates elicited by DNA damage responses may impact aging and age-associated diseases, it is important to understand the mechanisms governing DNA-damage associated cell-fate decisions.

It is possible that the loss of homeostasis between signaling networks affects cellular outcomes downstream of DNA damage responses, which would suggest that there are critical signaling thresholds determined by the level of DNA damage. For example, relatively high levels of DNA damage activate signaling pathways that regulate cell survival and apoptosis [5]. However, it is less clear how cell-fate decisions are made in cells exposed to chronic levels of DNA damage. Because individual cells must make cell-fate decisions under physiological and genotoxic conditions to maintain organismal homeostasis, it is important to determine how cells respond to the persistent induction and accumulation of DNA damage.

Here we exposed cultured cells or mice to various quantities and qualities of ^137^Cs γ-irradiation. Automated fluorescence microscopy was used to monitor effects of this irradiation on numerous human cell types. This experimental system allowed us to quantitatively assess the dynamic behavior of cells exposed to a wide range of DNA damage, providing insights into cell-fate decisions that are determined by the dose rate of chronic γ-irradiation.

## Materials and Methods

### Cell lines and cell culture conditions

Primary human fibroblasts (passage 9, NHDF p9) [6], TIG-3 primary human fibroblast (passage 27, TIG-3 p27) [7], and the immortalized MRC-5/hT cells [7], TIG-3/hT cells [7] and BJ1/hT cells [8] transfected with hTERT were maintained in minimum essential medium eagle alpha modification (Sigma) supplemented with l-glutamine and 10% fetal bovine serum (FBS). Five human tumor cell lines obtained from ATCC, MCF-7 (a mammary carcinoma cell line), U2OS (an osteosarcoma cell line), Saos-2 (an osteosarcoma cell line), HCT-116 (a colorectal carcinoma cell line), and HeLa (a cervical carcinoma cell line) were cultured in minimum essential medium eagle alpha modification or McCoy's 5A medium (Sigma) supplemented with l-glutamine and 10% FBS. A spontaneously immortalized breast epithelial cell line (MCF10A) (ATCC) was cultured in mammary epithelium basal medium (Lonza) with supplements. All cells were maintained in a humidified 5% CO_2_ atmosphere at 37°C. To optimize γ-irradiation conditions at different dose rates (0.007–0.694 mGy/min), cell culture incubators were placed at different distances from a ^137^Cs radiation device (1.11 TBq) (Sangyo Kagaku). The dose rate associated with each incubator was measured using a GD-302M glass dosimeter (AGC Techno Glass). ATM kinase activity was inhibited by incubating cells with 10 µM KU55933 (Merck Millipore). DNA-PKcs kinase activity was inhibited by incubating cells with 10 µM NU7026 (Merck Millipore).

### Colony formation and clonogenic survival assay

Colony formation assays with chronic γ-irradiation were performed by plating 100 or 200 cells in 60-mm culture dishes. Cells were cultured at different dose rates (0.007–0.694 mGy/min) for indicated amounts of time and then allowed to form colonies for 6–10 days. Cells were stained with crystal violet. The sensitivity of cells to ionizing radiation was measured using a clonogenic survival assay as described [9]. Briefly, for acute irradiation 1–100×10^2^ cells were seeded into 60-mm culture dishes and irradiated with a γ-ray dose that ranged from 1 to 5 Gy (^137^Cs source, 148 TBq, Gammacell 40 Exactor, Best Theratronics). After 10–14 days of incubation, colonies were stained with crystal violet and counted. Only colonies containing ≥50 cells were scored as survivors. Survival fractions were calculated in each experiment as the average cloning efficiency after treatment from at least two parallel dishes. Survival fractions were corrected for plating efficiency.

### Immunofluorescence staining and automated fluorescence microscope analysis

Cells plated on 96-well plates (μ-Plate, ibidi) were fixed with 4% paraformaldehyde in PBS for 30 min at room temperature, and then treated with 0.5% Triton X-100 in PBS for 20 min. Cells were immunolabeled using antibodies against TP53BP1 (1∶20,000; BD Biosciences) or γ-H2AX (1∶25,000; Millipore) and secondary antibodies conjugated with Alexa fluor 555 (1∶2000; Life Technologies). Nuclei were labeled with Hoechst 33258. Fluorescence images were obtained using an automated fluorescence microscope (IN Cell Analyzer 2000; GE Healthcare BioScience). The number of TP53BP1 foci per cell was determined using the image-analysis software IN Cell Developer (GE Healthcare BioScience).

### Cell cycle analysis

To analyze cell-cycle distributions, cells in S-phase were stained with EdU using a Click-It EdU Alexa Fluor 488 imaging kit (Life Technologies). Briefly, EdU was added to the growth medium (10 µM final concentration) for 30 min. Cells were then fixed with 4% paraformaldehyde, and labeled with a Click-It cocktail containing Alexa Fluor 488 azide and Hoechst dye. Fluorescence images of nuclei were obtained using automated fluorescence microscopy. Cell-cycle distribution analyses were performed using IN Cell Developer software.

### Mice and γ-irradiation

Female C57BL/6N mice that were 8–12 weeks old were purchased from Kyudo Co. (Saga, Japan). Mice were housed 5–10 animals per cage under specific pathogen-free conditions at 55–60% relative humidity and 22±2°C. Mice were irradiated using a Gammacell 40 Exactor or in a chronic ^137^Cs γ-irradiation area, as described for the cell culture experiments. The dose rate for the acute exposure was 1 Gy/min and dose rates for chronic exposures were 0.347, 0.694, or 1.388 mGy/min. Mice lacking *TP53* were obtained as described [10], [11]. Female wild-type and *TP53*-null mice that were 8–12 weeks old were irradiated as described above. All mice were maintained according to Guiding Principles for the Care and Use of Animals. All experiments were approved in advance by the Ethics Committee of Animal Care and Experimentation at the University of Occupational and Environmental Health, Kitakyushu, Japan (Admission Number: AE04-047). All surgery was made to minimize suffering. Isolation of primary lung cells from irradiated mice were performed as described previously [12] with some modification. In briefly, the mice were sacrificed under diethyl ether anesthesia. After collection with lung tissue to tubes containing DMEM/F12 (Gibco)/10% FBS, lung tissue were minced with a sterile scalpel on a 100 mm-dish for about 3 min, then transferred into tubes filled with collagenase/hyaluronidase (Stem Cell Technologies)/Epicult medium (Stem Cell Technologies)/5% FBS and incubated under constant agitation in a shaker for 1 h at 37°C. After centrifugation, collected pellets were treated with pre-warmed trypsin/EDTA (Gibco) for 1 min at 37°C, mixed the content of the tubes up and down for at least 1 min. After centrifugation, added 5 ml of pre-warmed dispase (Stem Cell Technologies)/DNase and incubated for 1 min at 37°C. After centrifugation, added ammonium chloride (Stem Cell Technologies), centrifugated again, after adding DMEM/F12 medium, mononuclear cells were isolated by filtering suspension through a 40 µm mesh.

### siRNA transfection

siRNA transfections were performed using Lipofectamine RNAiMAX (Life Technologies). TP53-specific siRNAs (VHS40366, s606, s607), P21-specific siRNAs (s416, s417), and non-targeting siRNA negative controls (AM4635, 4390846) were purchased from Life Technologies (Stealth RNAi or Silencer Select validated siRNAs).

### Western blot analysis

Western blot analysis was performed using 10 µg of whole cell extracts as described [9], with modifications. Proteins were electrophoresed on a 5–20% sodium dodecyl sulfate polyacrylamide gradient gel (Atto). Blots were labeled using primary antibodies against TP53 (1∶1000, Ab-6; Merck Millipore), phospho-TP53-Ser15 (1∶1000; Cell Signaling), p21 (1∶1000; BD Biosciences), MDM2 (1∶200, SMP-14; Santa Cruz Biotech), MDMX/HDMX (1∶5000; Bethyl), CHEK2 (1∶1000; Cell Signaling), phospho-CHEK2-Thr68 (1∶1000; Cell Signaling), β-tubulin (1∶1000; Sigma-aldrich), and β-actin (1∶10,000; Sigma-aldrich).

### Micronucleus assay

Irradiated cells in 96-well plates were fixed with 100% methanol at −20°C. Nuclei were stained with Hoechst 33258 and cytoplasms were visualized with the SYTO RNASelect green fluorescent cell stain (Life Technologies). Fluorescence images were obtained using an IN Cell Analyzer 2000. Micronuclei frequency was determined using In Cell Developer software.

### Senescence detection assay

Irradiated cells were replated in triplicate in 6-well plates (1×10^4^ cells per well) and cultured for 24 h in 5% CO_2_ at 37°C. Senescence-associated β-gal activity was detected with a senescence detection kit (K320-250, Biovision). More than 300 cells per sample were counted to determine the percentage of senescent cells.

## Results

### Proliferating human fibroblasts are particularly sensitive to chronic γ-irradiation

**Figure 1:**
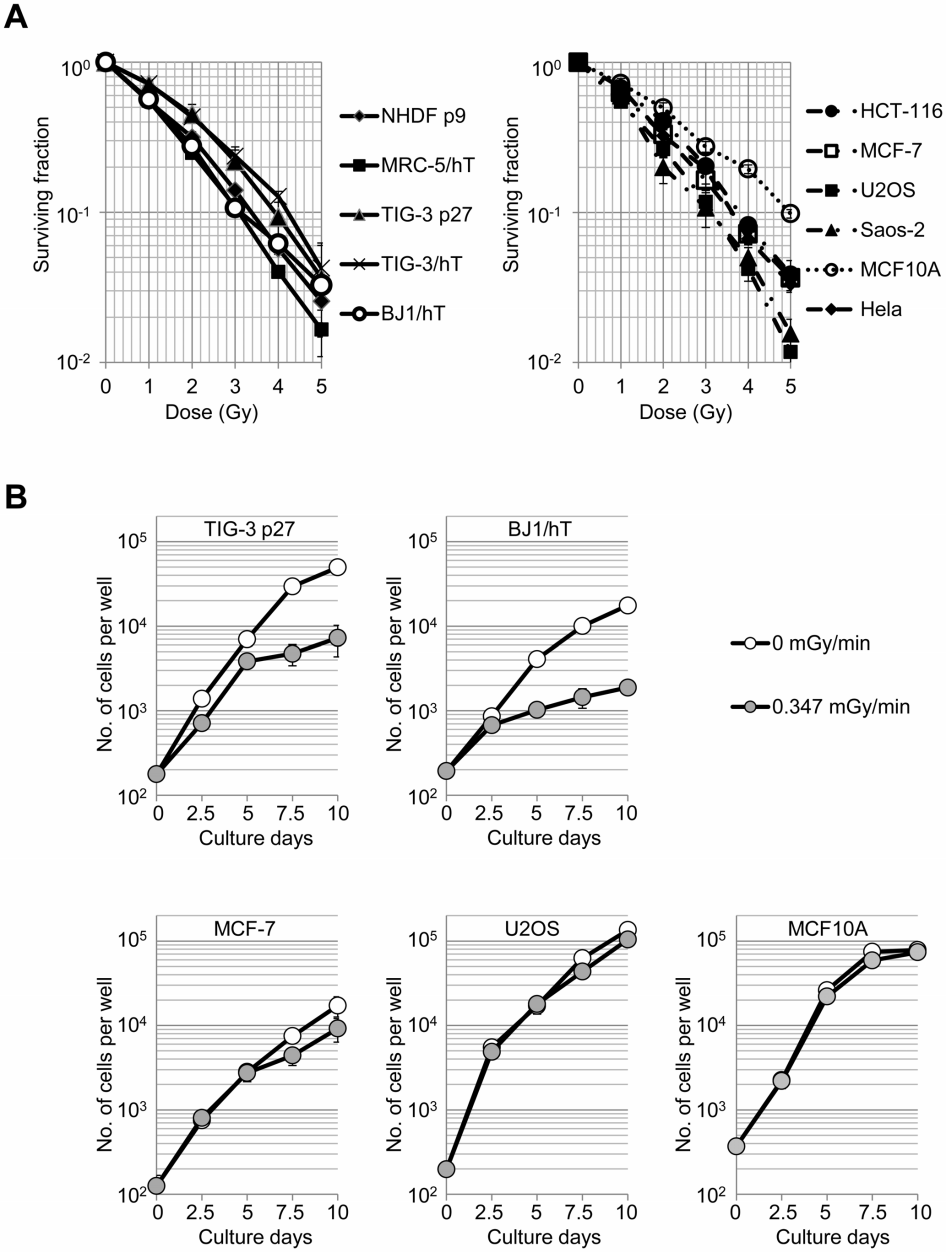
Different exposure conditions result in different sensitivities of proliferating cells to ionizing radiation. (A) Clonogenic survival of various human cell lines exposed to acute ^137^Cs γ-irradiation (1 Gy Gy/min). Surviving fractions are expressed relative to the plating efficiencies of non-irradiated cells. Values represent the mean ± SD of three independent experiments. (B) Cell proliferation of various human cell lines under chronic γ-irradiation conditions. Cells exposed to 0.347 mGy mGy/min (grey circles) are compared to unirradiated controls (white circles).

It is generally accepted and well discussed that different types of cells exhibit different sensitivities to ionizing radiation, depending on the cell's origin, differentiation status, and genetic background [13], [14]. For example, cells from patients with Ataxia-telangiectasia or Nijmegen breakage syndrome are particularly sensitive to ionizing radiation because a protein required for DNA damage responses is dysfunctional in these patients [15]. In this study, all types of cultured cells that we examined (human primary fibroblasts, telomerase reverse transcriptase (TERT)-immortalized fibroblasts, immortalized cell lines, and tumor cell lines) showed different, but relatively comparable, sensitivity to acute γ-irradiation exposure at a dose rate of 1.0 Gy/min (Figure 1A). The D_0_ values (37% survival dose) were between 1.1 and 1.4 Gy. Individual cells treated with acute γ-irradiation determine an alternative cell fate at several decision points after massive and transient DNA damage [16]. Sustained DNA damage also causes individual cells to adopt a wider range of cell fates, including apoptosis, DNA-damage tolerance, or proliferation. In this context, the rates of induction and accumulation of DNA damage theoretically affect these cell-fate decisions. We therefore hypothesized that cells could tolerate low levels of DNA damage (chronic γ-irradiation) and would exhibit specific traits depending on their radiation sensitivity.

To test this hypothesis, we placed cell-culture incubators at different distances from a ^137^Cs radiation source, allowing us to expose cells to chronic γ-irradiation at a wide range of dose rates (Figure S1A). We first exposed a number of cultured cell types to chronic γ-irradiation at a dose-rate of 0.347 mGy/min for 10 days (0.5 Gy/day, or 5 Gy in total). Cells were then analyzed for their colony forming ability. In contrast to results obtained with acute γ-irradiation, colony formation following chronic γ-irradiated was cell-type specific. Chronic γ-irradiation at a dose rate of 0.347 mGy/min had very little effect on tumor cell lines compared with unirradiated controls. Human fibroblasts, however, showed marked decreases in the number and size of macroscopic colonies (both primary and *hTERT*-immortalized fibroblasts; Figure S1B and Table 1).

**Table 1:** **Comparison of colony forming abilities of different types of cells for acute versus chronic γ-irradiation (5 Gy).**

Cell line	Surviving fraction^1^ for acute γ-irradiation^2^	Surviving fraction for chronic γ-irradiation^3^
NHDF p9	0.026±0.003	0.069±0.015
BJ1/hT	0.033±0.005	0.041±0.013
TIG-3 p27	0.037±0.026	0.119±0.038
MCF10A	0.094±0.006	0.643±0.055
HCT-116	0.039±0.009	0.942±0.015
MCF-7	0.037±0.005	0.914±0.027
U2OS	0.012±0.003	0.933±0.010
Hela	0.033±0.003	0.924±0.068

Values represent the mean ± SD of three independent experiments.

1 Survival fractions were determined based on crystal violet staining.

2 Dose rate of 1.0 Gy/min for 5 min, total dose; 5 Gy. Cells were cultured for 10 days following acute γ-irradiation.

3 Cells were cultured for 10 days at dose rate of 0.347 mGy/min, total dose; 5 Gy.

To test whether insensitivity to low-dose irradiation was specific to transformed cells, we examined a spontaneously immortalized but non-transformed breast epithelial cell line (MCF10A). Similar to the cancer cell lines, MCF10A cells were not sensitive to chronic γ-irradiation at a dose rate of 0.347 mGy/min. When 8 cell lines were exposed to 5 Gy of γ-irradiation under acute or chronic conditions, all cells were sensitive to acute exposure, but only fibroblasts were affected by chronic exposure (Table 1). We next used automated fluorescence microscopy to assess the proliferation of different cell types exposed to chronic γ-irradiation (0.347 mGy/min). Consistent with colony-formation results, the proliferation of fibroblasts (TIG-3 p27, and BJ1/hT) was reduced compared to unirradiated controls, whereas other cell lines (MCF-7, U2OS, MCF10A) were not affected (Figure 1B). These results reveal a distinct sensitivity of human fibroblasts to chronic γ-irradiation.

### Chronic γ-irradiation produces distinct effects on fibroblasts in a dose rate-dependent manner

**Figure 2:**
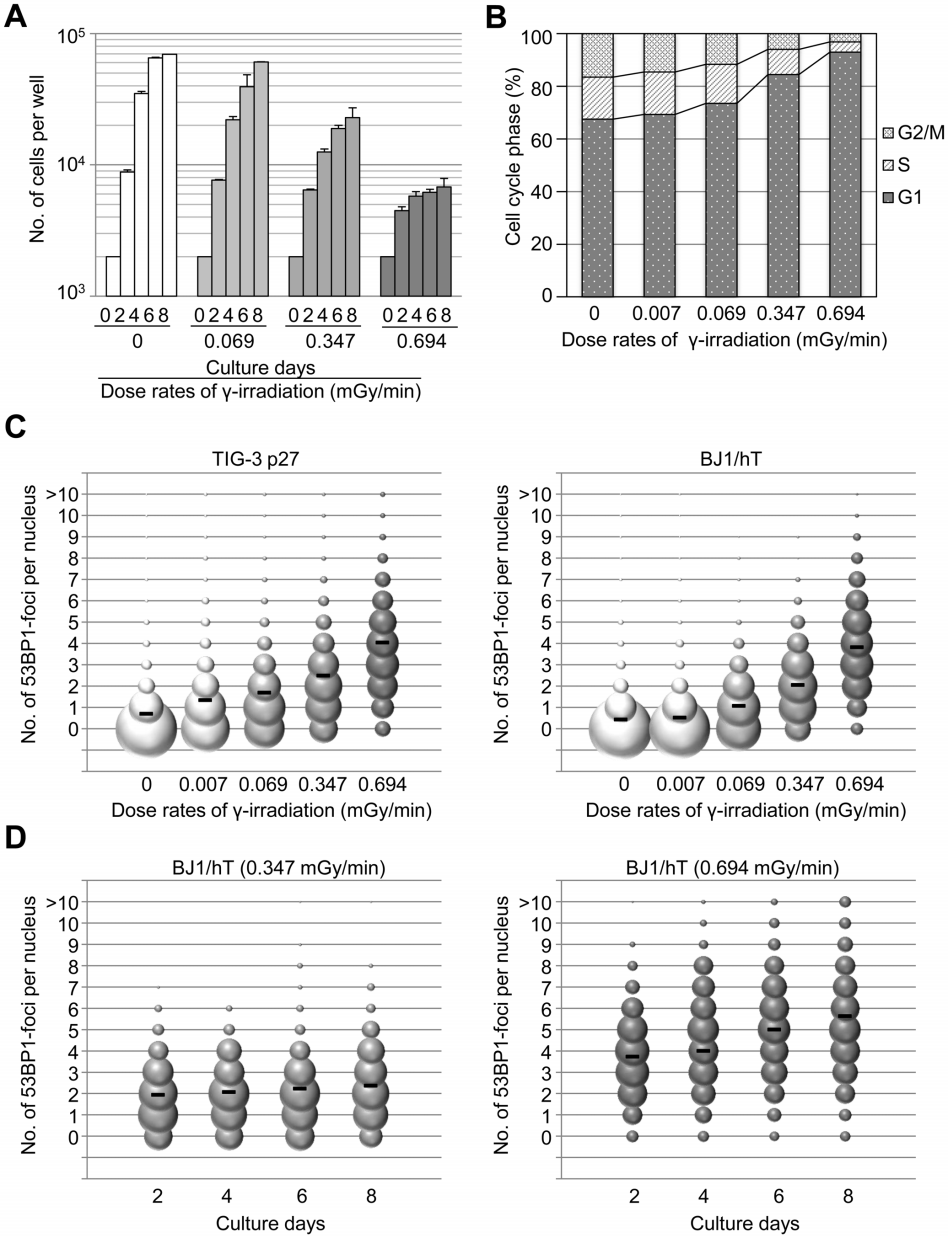
Chronic γ-irradiation suppresses the proliferation of human fibroblasts by inducing a G1 cell-cycle arrest. (A) Dose-dependent effect of chronic γ-irradiation on the proliferation of primary human fibroblasts (TIG-3 p27 cells). Values represent the mean ± SD of three independent wells. (B) Representative cell cycle distribution of TIG-3 p27 cells after 4 days of chronic γ-irradiation. (C) The frequency distribution of DNA damage-associated TP53BP1-foci in TIG-3 p27 (left) or BJ1/hT (right) cells exposed to 4 days of chronic γ-irradiation. At least 1×10^3^ cells per well were examined to determine the frequency distribution. The size of the bubble is proportional to the number of cells with that number of TP53BP1-foci. Black bars indicate the mean number of TP53BP1-foci per cell. (D) Time-course analysis of TP53BP1 foci in BJ1/hT cell exposed to 0.347 (left) or 0.694 (right) mGy/min of chronic γ-irradiation.

To characterize the sensitivity of human fibroblasts to chronic γ-irradiation, we used automated fluorescence microscopy to quantitatively analyze dose rate-dependent effects. Cells were exposed to different dose rates of chronic γ-irradiation (0, 0.069, 0.347, or 0.694 mGy/min) for ≤10 days. The proliferation of human fibroblasts decreased in a dose rate-dependent manner (Figure 2A). Again, this effect was significant in human fibroblasts, as the proliferation of epithelial cells was less affected by chronic irradiation (Figure S2A). To determine the cell-cycle distribution of these fibroblasts, nuclei were labeled with Hoechst and the S-phase maker 5-ethynyl-2′-deoxyuridine (EdU). Under chronic γ-irradiation conditions, there were fewer mitotic cells but no increase in dead cells (Figure S2B), suggesting that cells were arrested in a slow- or non-cycling state. Furthermore, cells arrested in the G1 phase of the cell cycle in a dose rate-dependent manner (Figure 2B).

We next sought to determine whether different dose rates caused different levels of DNA-damage accumulation. Cells were exposed to chronic γ-irradiation for 4 days and then the number of tumor protein p53 binding protein 1 (TP53BP1) foci, which indicates DNA damage, was counted within each nucleus. In both TIG-3 p27 and BJ1/hT cell lines, chronic γ-irradiation caused a dose rate-dependent increase in TP53BP1 foci per nucleus (Figure 2*C*). Similar results were obtained when foci of phosphorylated histone H2AX (γ-H2AX), which is another marker of DNA damage, were analyzed (Figure S3A). Given that DNA damage can be repaired by a number of pathways, it was interesting that different dose rates caused different levels of DNA damage. We therefore sought to determine the irradiation dose rate that induced the accumulation of DNA damage. Cells were exposed to chronic γ-irradiation at dose rates of 0.347 or 0.694 mGy/min and the number of TP53BP1-foci was measured every 2 days. There was a clear difference in the level of DNA damage between these two dose rates. At 0.694 mGy/min, but not 0.347 mGy/min, we observed increased numbers of TP53BP1 and γ-H2AX foci in a time-dependent manner (Figure 2D and S3B). These data indicate that DNA damage begins to accumulate in cultured human fibroblasts when they are exposed to between 0.347 and 0.694 mGy/min of chronic γ-irradiation.

### γ-irradiated cells undergo senescence when the dose rate exceeds the threshold for DNA-damage accumulation

**Figure 3:**
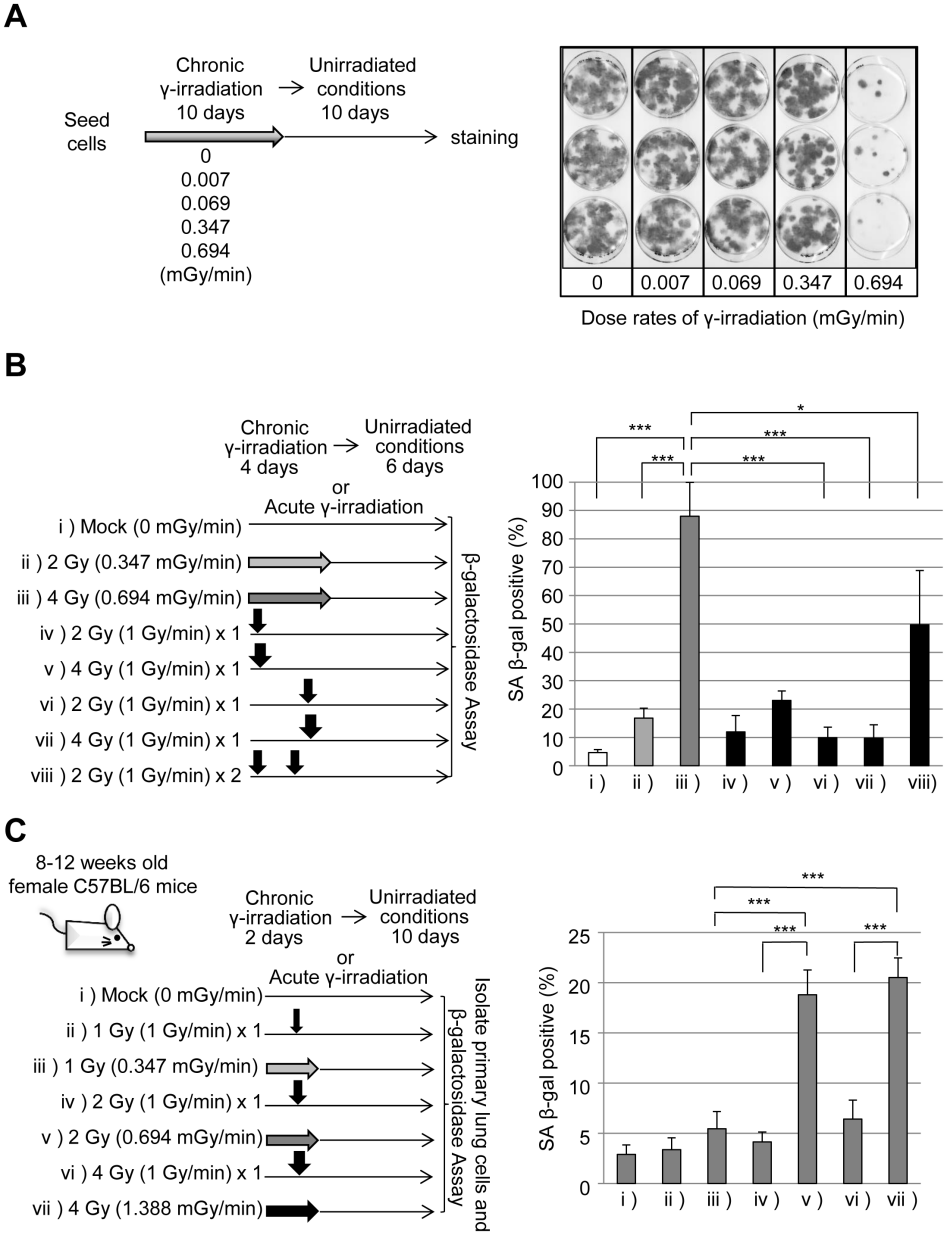
The dose rate of chronic γ-irradiation affects cell-fate decisions. **(A) Colony-forming ability of fibroblasts following chronic γ-irradiation.** The experimental scheme is illustrated to the left. TIG-3 p27 cells (2×10^2^) were exposed to different dose-rates of γ-irradiation for 10 days and then allowed to grow under unirradiated conditions for an additional 10 days. Representative pictures of crystal violet-stained colonies are shown for each dose rate. (B) Cellular senescence induced by acute or chronic γ-irradiation. BJ1/hT cells (4×10^3^) were exposed to the treatment condition indicated on the left. Grey arrows indicate chronic γ-irradiation at indicated dose rates. Black arrows indicate acute γ-irradiation of indicated doses. Following 1 day of recovery and 6 days of additional growth, cells were stained for β-gal activity to assess levels of senescence (right). Values represent the mean ± SD of three independent experiments. *P<0.05, ***P<0.001. (C) Cellular senescence induced by acute or chronic γ-irradiation *in vivo*. Female C57BL/6 mice were exposed to the treatment condition indicated to the left. Grey arrows indicate chronic γ-irradiation at indicated dose rates. Black arrows indicate acute γ-irradiation of indicated doses. Primary cells isolated from lungs of irradiated mice were stained for β-gal activity to assess levels of senescence (right). Values represent the mean ± SD of independent cultures from 2 mice. ***P<0.001.

We next wondered whether 0.347 and 0.694 mGy/min chronic γ-irradiation dose rates elicited different cellular responses. In particular, we sought to determine whether different dose rates resulted in reversible or irreversible cell-cycle arrest. Cells were exposed to different dose rates of γ-irradiation for 10 days, and then cultured for an additional 10 days (free from irradiation) to determine whether they could re-enter the cell cycle. Cells exposed to ≤0.347 mGy/min formed colonies during the irradiation-free culture period, but cells exposed to 0.694 mGy/min exhibited severe growth impairment (i.e., they were senescent; Figure 3A). In this experiment, differences between 0.347 and 0.694 mGy/min did not result from different total irradiation exposures because the same result was obtained when exposure times were adjusted to equalize the total irradiation dose (Figure S4A and B). When analyzed immediately following the irradiation exposure, cells exposed to 0.347 mGy/min arrested in G1 and did not form well-defined colonies (Figure S1B and S4C), but could re-enter the cell cycle when subsequently cultured in irradiation-free conditions. In contrast, cells exposed at 0.694 mGy/min could not form colonies following irradiation exposure suggesting that they had adopted an irreversible senescence-like growth arrest. We tested whether the 0.694 mGy/min dose rate induced senescence by measuring β-galactosidase (β-gal) activity [17]. Chronic γ-irradiation efficiently induced cellular senescence (i.e., β-gal activity) and 0.694 mGy/min was more potent than 0.347 mGy/min in this assay (Figure 3B). This result was consistent with results obtained using the colony survival assay (Figure 3A). Chronic irradiation at 0.694 mGy/min was considerably more effective than acute irradiation in inducing cellular senescence (Figure 3B).

To test the biological relevance of these results, we repeated these analyses in mice. Animals were irradiated with acute (1 Gy/min) or chronic (0.347–1.388 mGy/min) γ-rays and allowed to recover for 10 days (Figure 3C). Primary lung cells were then isolated from irradiated mice and stained for β-gal activity. At dose rates ≥0.694 mGy/min, chronic irradiation was more effective than acute irradiation in inducing senescence (given equal total doses; Figure 3C), thus confirming the in vitro data.

### The ATM/TP53/p21 pathway inhibits cellular growth in response to chronic γ-irradiation

**Figure 4:**
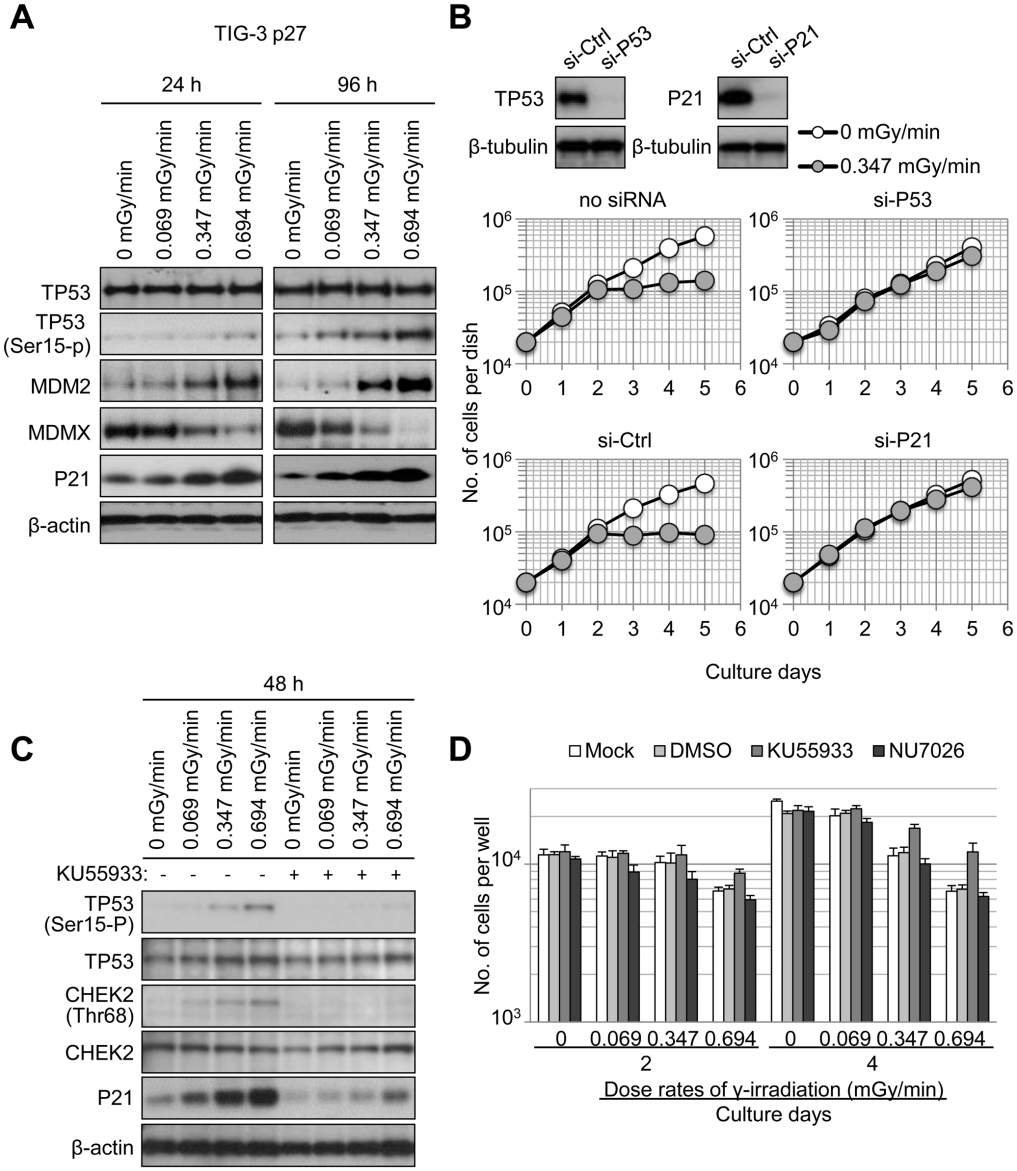
The ATM/TP53/p21 pathway mediates the effect of chronic γ-irradiation on cell proliferation. (A) Western blotting was used to determine levels of TP53, phosphorylated TP53 (Ser-15), MDM2, MDMX, and p21 in human fibroblasts (TIG-3 p27) cultured under chronic γ-irradiation conditions for 24 or 96 hours (dose rates are indicated). β-actin served as a loading control. (B) Knockdown of TP53 or p21 in BJ1/hT cells abolishes the growth inhibitory effect of chronic γ-irradiation. Western blot analysis of cells transfected with control siRNA (si-Ctrl), TP53-specific siRNA (si-P53), or P21-specific siRNA (si-P21) are shown (top). β-tubulin served as a loading control. Whole cell lysates were prepared 48 hours after the transfection. Forty hours after the transfection. Forty-eight hours after transfection, BJ1/hT cells were placed into culture and grown under unirradiated control conditions (white circles) or a γ-irradiation dose rate of 0.347 mGy mGy/min (grey circles). The number of cells per dish was counted after indicated periods of time. (C) ATM kinase is activated in response to chronic γ-irradiation. BJ1/hT cells were exposed to indicated dose rates of chronic γ-irradiation in the presence or absence of the ATM kinase inhibitor KU55933 (10 µM). ATM kinase activity was assessed by detecting phosphorylated TP53 (Ser15) or phosphorylated CHEK2 (Thr68) by Western blotting. TP53 activation and P21 induction by chronic γ-irradiation were examined by Western blotting. (D) Inhibition of ATM kinase, but not DNA-PKcs, attenuates the growth inhibitory effect of chronic γ-irradiation. BJ1/hT cells were cultured under chronic γ-irradiation conditions at indicated dose rates in the presence or absence of KU55933 (10 µM) or the DNA-PKcs inhibitor NU7026 (10 µM). Values indicate the mean ± SD of three independent wells.

The tumor suppressor protein p53 (TP53) regulates cell fate following DNA damage [18]. We therefore hypothesized that TP53 was involved in the G1 cell-cycle arrest induced by chronic γ-irradiation. To test this hypothesis, the expression of TP53 (and related proteins) in irradiated cells was analyzed by western blotting. Although TP53 levels were not affected by γ-irradiation (even at 0.694 mGy/min), γ-rays resulted in the phosphorylation of TP53 (at Ser 15), and increased levels of p21 and MDM2 oncogene, E3 ubiquitin protein ligase (MDM2), which are direct transcriptional targets of TP53 [19] (Figure 4A). In addition, MDMX, which negatively regulates TP53, was down-regulated in a dose rate-dependent manner. This result is consistent with a DNA damage response [9]. Notably, these inductions persisted for 96 hours, indicating sustained TP53 activity in response to chronic γ-irradiation. Interestingly, in other tumor cells also exhibit similar expression patterns in same proteins, but seem to be relatively insensitive especially when cells are irradiated at a dose-rate of 0.347 mGy/min (Figure S5).

To investigate the role of TP53 in cellular responses to chronic γ-irradiation, TP53 or p21 levels were knocked down in BJ1/hT fibroblasts. This treatment abolished the cell-proliferation inhibition induced by chronic γ-irradiation (0.347 mGy/min; Figure 4B). These results were confirmed in TIG-3 p27 cells (Figure S6A) and imply that the TP53/p21 pathway is required for chronic γ-irradiation-induced inhibition of cell proliferation in fibroblasts. Ataxia telangiectasia mutated (ATM) mediates cellular responses to ionizing radiation. In particular, ATM is involved in DNA-repair signaling, regulates multiple cell-cycle checkpoints, and acts upstream of TP53 following DNA damage [20]. We therefore asked whether inhibition of ATM affected the response of fibroblasts to chronic γ-irradiation. The ATM inhibitor KU55933 inhibited the phosphorylation of TP53 and checkpoint kinase 2 (CHEK2) (ATM kinase substrates) and reduced p21 induction in response to γ-irradiation (Figure 4C). In addition, KU55933 restored levels of cell proliferation in chronic γ-irradiated cells (Figure 4D for BJ1/hT cells and Figure S5B for TIG-3 p27 cells). In addition to ATM, DNA-PKcs also senses DNA damage and non-homologous end-joining repair of double-strand breaks [21]. However, the DNA-PKcs inhibitor NU7026 did not rescue proliferation of cells exposed to chronic γ-irradiation cells compared with mock- or DMSO-treated controls (Figure 4D for BJ1/hT cells and Figure S6B for TIG-3 p27 cells). These data indicate that ATM, but not DNA-PK, modulates cellular responses to DNA damage that are induced by chronic γ-irradiation.

### The absence of TP53 abolishes chronic γ-irradiation-induced cellular senescence

**Figure 5:**
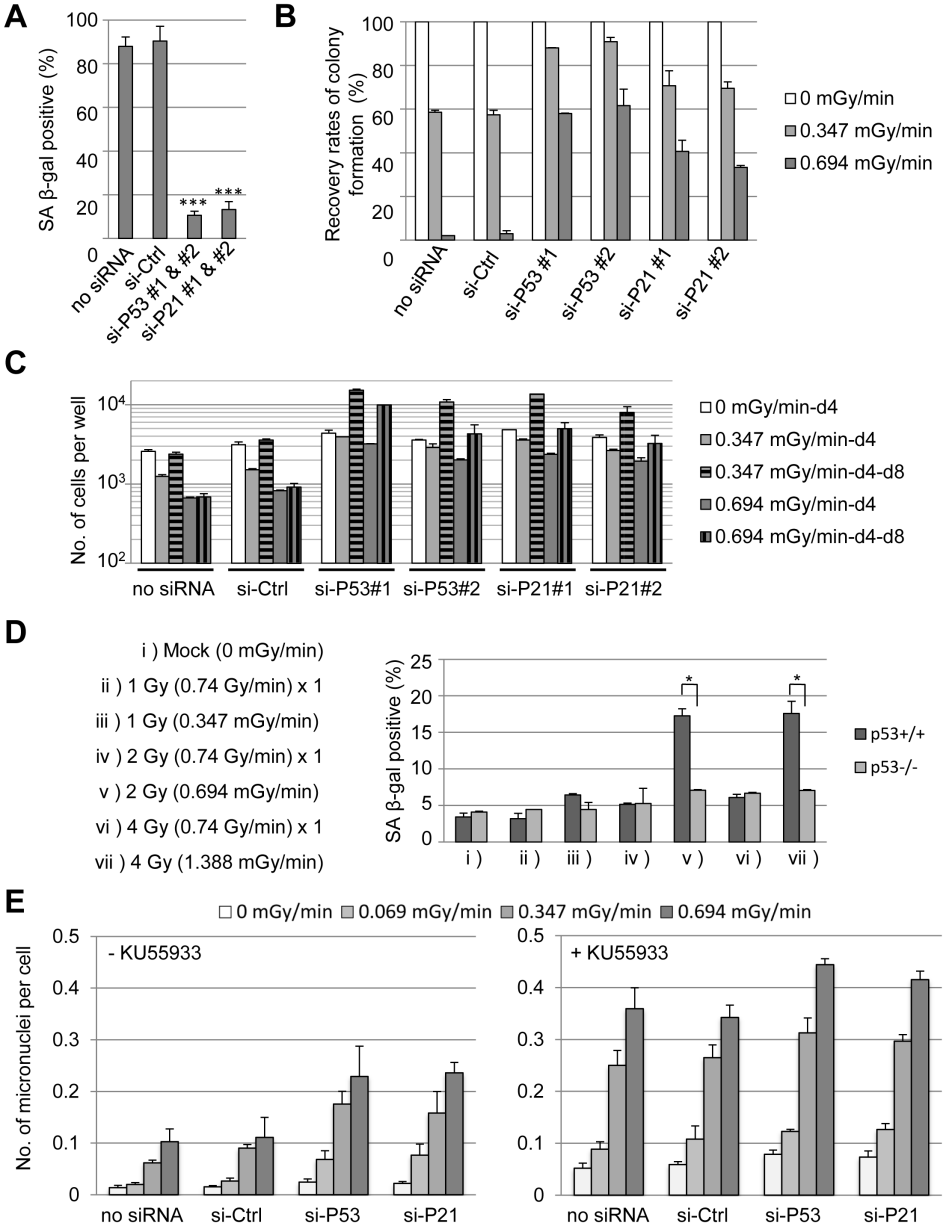
The ATM/TP53/p21 pathway maintains genomic integrity under chronic γ-irradiation conditions by regulating cell-fate decisions. (A) BJ1/hT cells were transfected with indicated siRNAs and cultured for 4 days under chronic γ-irradiation conditions (0.694 mGy mGy/min). β-gal activity was used to assess cellular senescence. Values represent the mean ± SD of three independent experiments. ***P<0.001. (B) BJ1/hT cells were transfected with indicated siRNAs and then cultured under chronic γ-irradiation conditions at indicated dose rates for 4 days. After an additional 10 days of growth in unirradiated conditions, the number of macroscopic colonies was determined. Values were normalized to control and represent the mean ± SD of three independent experiments. (C) BJ1/hT cells were transfected with indicated siRNAs and then cultured under chronic γ-irradiation conditions at indicated dose rates for 4 days (-d4). Some cells were cultured an additional 4 days under unirradiated conditions (-d4-d8). Values represent the mean ± SD of three independent wells. (D) Wild-type or *TP53* null mice were exposed to acute (ii, iv, vi) or chronic (iii, v, vii) γ-irradiation treatment conditions, as indicated on the left. β-gal activity was used to assess cellular senescence of primary lung cells isolated from these mice. Values represent the mean ± SD of independent cultures from 2 mice. *P<0.05. (E) BJ1/hT cells were transfected with indicated siRNA and then cultured without (left) or with (right) KU55933 (10 µM) under chronic γ-radiation conditions at indicated dose rates for 4 days. The number of micronuclei was then assessed. Values represent the mean ± SD of at least three independent experiments.

Our data indicate that the dose rate of chronic γ-irradiation (in combination with the rate of DNA damage) affects cellular responses of fibroblasts (e.g., DNA-damage tolerance, reversible G1 cell-cycle arrest, or irreversible senescence). As the TP53/p21 pathway was required for growth arrest in response to chronic γ-irradiation at 0.347 mGy/min (Figure 4B and Figure S6A), we asked whether TP53 or p21 were also essential for the induction of senescence at 0.694 mGy/min. Cells in which TP53 or p21 was knocked down were irradiated for 4 days and then analyzed for β-gal activity. Loss of TP53 or p21 reduced the number of β-gal-positive cells (Figure 5A). This data demonstrate that the TP53/p21 pathway is also essential for cellular senescence in response to chronic γ-irradiation. To confirm this result, colony formation and regrowth following 4 days of γ-irradiation were assessed for the two dose rates (Figure 5B and C). When BJ1/hT cells were exposed to chronic γ-irradiation at 0.694 mGy/min, knockdown of TP53 or p21 restored colony formation and regrowth compared to controls (Figure S7A). Similar results were observed for TIG-3 p27 cells (Figure S7B–D). To determine whether the TP53 pathway is essential for the induction of cellular senescence following chronic γ-irradiation *in vivo*, wild-type and *TP53* null mice were irradiated at different doses and dose rates (Figure 3C) and levels of senescence were determined for primary cultured cells. Chronic γ-irradiation increased levels of senescence in wild-type cells but not in cells lacking *TP53* (Figure 5D). Thus, the TP53 pathway regulated cell-fate responses to chronic γ-irradiation both *in vitro* and *in vivo*.

Finally, we examine the effect of the ATM/TP53/p21 pathway on genomic stability in fibroblasts exposed to chronic γ-irradiation. Genomic stability was assessed by measuring the frequency of micronucleus formation [22]. Fibroblasts transfected with *TP53*- or *p21*-specific siRNA were exposed to chronic γ-irradiation in the presence or absence of an ATM inhibitor for 4 days. Knockdown of TP53 or p21 increased the number of micronuclei compared to controls (Figure 5E), suggesting that the TP53/p21 pathway helps to maintain genomic stability in response to chronic γ-irradiation. In addition, ATM inhibition elevated the frequency of micronucleus formation in response to chronic γ-irradiation and abolished the difference between control and TP53/p21 deficient cells (Figure 5E). Thus, ATM is epistatic to the TP53/p21 pathway concerning cell-fate decisions in response to chronic γ-irradiation.

## Discussion

DNA damage induced by ionizing radiation can significantly impact cell fate, i.e., affect whether a cell will proliferate, differentiate, become senescent, or apoptosis, for example [23], [24]. In general, DNA-damage-associated cell-fate decisions are determined by a cell's properties (e.g., cell type and tissue of origin) [25]. In addition to cellular properties, the quantity and quality of DNA damage also affect these cell-fate decisions. The dose rate of sparsely ionizing radiation is particularly important, as lower dose rates produce smaller biological effects, even when the total dose is held constant [26]. The physiological properties of cells, including the abilities to sense, repair, and tolerate DNA damage, are likely important determinants of fate when cells are exposed to different rates of DNA damage. In this study, we tested whether the rate of DNA damage affects cell-fate decision in proliferating cells.

We discovered that different cell types exhibited different susceptibilities to chronic γ-irradiation, whereas these same cell types responded similarly to acute γ-irradiation. We defined susceptibility to γ-irradiation as the inability of exposed cells to form a colony, i.e., cell death or senescence. However, when a population of cells is exposed to acute γ-irradiation cells within a single colony can exhibit quite heterogeneous behaviors [27], which may result from stochastic cellular responses to lethal and sub-lethal amounts of DNA damage. Interestingly, fibroblasts were most susceptible to chronic γ-irradiation. Susceptibility in this context was defined as a G0/G1 cell-cycle arrest, which is regulated by a number of cell-signaling pathways. Therefore, depending on the frequency of DNA damage and the period of exposure, cell-fate decisions are deterministic, rather than stochastic.

Here we discovered that two γ-irradiation dose rates induced different responses in fibroblasts, namely reversible or irreversible cell-cycle arrest. Precise regulation of proliferation and quiescence/senescence is essential for the development and homeostasis of multicellular organisms [28]–[30], and cellular stresses often promote cell-cycle exit [31]. A large number of proteins involved in cell-cycle progression and regulation have been identified, and complex interactions between these proteins during different cell-cycle stages and during checkpoint controls have been described [32], [33]. However, the molecular mechanisms underlying cell-fate decisions under different cellular statuses remain unclear. Interestingly, our experimental system allowed us to deliver γ-irradiation at different dose rates, and cellular responses to this treatment likely reflected the DNA damage response systems work together to maintain genomic integrity, and help to determine cell fate in response to DNA damage. However, it is unclear how these systems are coordinate because the molecular events activated by DNA damage are complex and dynamic.

The TP53 regulates cellular responses (e.g., cell-cycle arrest, senescence, and apoptosis) to genotoxic stresses, thereby suppressing cellular malignancy and maintaining homeostasis within multicellular organisms [34], [35]. We showed here that the TP53 pathway was a critical component of cellular responses to chronic γ-irradiation, and that these responses were triggered by the rate of DNA damage. Because TP53 regulates many physiological responses, the molecular mechanisms by which TP53 selectively induces sets of transcriptional targets to ensure appropriate cellular outcome have been extensively investigated [18]. We provide experimental evidences supporting the essential role of TP53 in cell-fate decisions to chronic γ-irradiation *in vivo*, using *TP53* null mice. There are small molecule TP53 inhibitors that are able to modify the activity of TP53 *in vivo* [36], and that also will be useful tool for further studies of TP53 functions in cell-fate decisions. TP53 and its negative regulator MDM2 form a tight autoregulatory feedback loop, which regulates TP53 stability and activity [37], [38]. ATM and wild type TP53-inducible phosphatase 1 (Wip1) phosphorylate and dephosphorylate TP53, respectively, thereby forming another autoregulatory feedback loop [39]. These feedback loops ensure that DNA damage-mediated TP53 activity is transient [40], [41]. However, our data indicate that these feedback loops also provide intrinsic tolerance to low levels of DNA damage. Overall cellular status and fate are dynamically regulated by nested signaling networks involving the TP53/MDM2 and ATM/Wip1 feedback loops [42]. A cell can respond to DNA damage by developing DNA-damage tolerance or by activating a DNA-damage response. Our data clearly demonstrated that the ATM/TP53/p21 pathway regulated the choice between DNA-damage tolerance and response when cells were exposed to chronic γ-irradiation and that this decision depended on the dose rate and the cell type. This decision is related to the balance between homeostasis and genomic stability, which is critical for the development and maintenance of viability of multicellular organisms [43]. It is therefore important to verify the hierarchy of the sensitive systems composed of the feedback loops in response to sustained low levels of DNA damage, as this information may shed light on the mechanisms of cancer and aging, for example. As we predicted, proliferating cells that are exposed to chronic γ-irradiation exhibit the most susceptible phenotype (cell-cycle arrest or senescence for fibroblasts) in response to the rate of DNA damage. Thus, we were able to distinguish between cell-fate decisions, allowing us to evaluate the mechanisms underlying this process. Future studies involving this system will provide insights into the molecular mechanisms governing cell-fate determination in response to DNA damage.

## Supporting Information

Figure S1 **Different cell types exhibit different radiation sensitivity when exposed to chronic γ-irradiation.** (A) A diagram of the culture room containing a ^137^Cs radiation source. Cubes indicate incubator positions. The dose rate for each incubator is indicated. (B) A variety of human cell lines were cultured for 10 days under control conditions (0) or chronic γ-irradiation conditions (0.347 mGy/min). Representative images of crystal violet-stained culture plates are shown.
(TIF)

Figure S2 **Chronic γ-irradiation specifically suppresses the proliferation of fibroblasts via a G1 arrest.** (A) A number of human cell lines were cultured under chronic γ-irradiation conditions at indicated dose rates. After 0, 2.5, 5, 7.5, or 10 days the number of cells in each well was determined. Values represent the mean ± SD of six independent wells. (B) Representative images of TIG-3 p27 cells after 4 days of chronic γ-irradiation (dose rates are indicated). Hoechst 33258 staining of DNA (blue) and EdU-Alexa Fluor488 (green) are shown. Scale bar is 250 µm.
(TIF)

Figure S3 **Human fibroblasts undergo senescence when accumulated DNA damage exceeds a threshold.** (A) BJ1/hT (left) or TIG-3 p27 (right) cells were cultured for 4 days under chronic γ-irradiation conditions at indicated dose rates. The number of γ-H2AX-foci per cell was then determined. The size of the bubble is proportional to the number of cells with that number of γ-H2AX-foci. Black bars indicate the mean number of foci per cell. (B) The number of γ-H2AX-foci increased over time in response to chronic γ-irradiation conditions. BJ1/hT (upper) or TIG-3 p27 (lower) cells were cultured under chronic γ-irradiation conditions at indicated dose rates for 2.5, 5, 7.5, or 10 days.
(TIF)

Figure S4 **The chronic γ-irradiation dose rate affects cell-fate decisions in human fibroblasts.** (A–C) Colony-forming ability of fibroblasts following chronic γ-irradiation. Experimental schemes are illustrated to the left. (A) TIG-3 cells (2×10^2^) were cultured at indicated dose rates for 5 days and then grown under unirradiated conditions for an additional 10 days. Culture plates were then stained using crystal violet. Representative images are shown to the right. (B) TIG-3 p27 cells (2×10^2^) were cultured for 10 days at 0.347 mGy/min or 5 days at 0.694 mGy/min (total dose of 5 Gy). Culture dishes were then incubated under unirradiated conditions for an additional 5 days and stained using crystal violet. Representative images of stained culture dishes are shown on the right. (C) TIG-3 p27 cells (2×10^2^) were cultured at indicated dose rates for 10 days. Representative images of crystal violet-stained culture dishes are shown to the right.
(TIF)

Figure S5 **TP53/p21 pathway is activated by chronic γ-irradiation in tumor cell lines.** Changes in protein levels of TP53, phosphorylation of TP53 at Ser-15, MDM2, MDMX, and P21 in U2OS and MCF-7 cells cultured under chronic γ-irradiation for 24 and 96 hours at different dose-rates of background 0, 0.069, 0.347, 0.694 mGy/min were analyzed by Western blotting.
(TIF)

Figure S6 **Inhibition of the ATM/TP53/p21 pathway attenuates chronic γ-irradiation induced growth inhibition.** (A) TIG-3 p27 cells (2×103) were transfected with indicated siRNAs and cultured under chronic γ-irradiation conditions at indicated dose rates. The number of cells per well was determined at indicated time points. Values represent the mean ± SD of three independent wells. (B) Inhibition of ATM kinase activity, but not DNA-PKcs activity, attenuates the growth inhibitory effect of chronic γ-irradiation. TIG-3 p27 cells were cultured for 2 or 4 days under chronic γ-irradiation conditions at indicated dose rates in the presence or absence of the ATM inhibitor KU55933 (10 µM) or the DNA-PKcs inhibitor NU7026 (10 µM). The number of Hoechst-stained nuclei was determined for each well. Values represent the mean ± SD of three independent wells.
(TIF)

Figure S7 **Knock down of TP53 or p21 attenuates chronic γ-irradiation-induced senescence.** (A) Western blot analysis of BJ1/hT cells transfected with control siRNA (si-Ctrl) or siRNA specific for *TP53* (si-P53 #1 or #2), or *P21* (si-P21 #1 or #2) (upper left). β-tubulin served as the loading control. Cells transfected with indicated siRNA were cultured under chronic γ-irradiation conditions at indicated dose rates for 4 days, and then incubated an additional 10 days under non-irradiated conditions (experimental scheme is illustrated lower left). Representative images of crystal violet-stained colonies are shown (right). (B) TIG-3 p27 cells were analyzed as in (A), except that cells were cultured for 6 days following γ-irradiation. (C–D) TIG-3 p27 cells transfected with indicated siRNAs were exposed to γ-irradiation at indicated dose rates. The ability of these cells to form colonies (C) or to proliferate (D) was subsequently assessed as shown in Figure 5 (B–C).
(TIF)
