# Supplementary figures and images for: A Novel ATM/TP53/p21-Mediated Checkpoint Only Activated by Chronic γ-Irradiation
Source: PLoS One. 2014 Aug 5;9(8):e104279. doi: 10.1371/journal.pone.0104279 (PMC4122452; doi:10.1371/journal.pone.0104279)

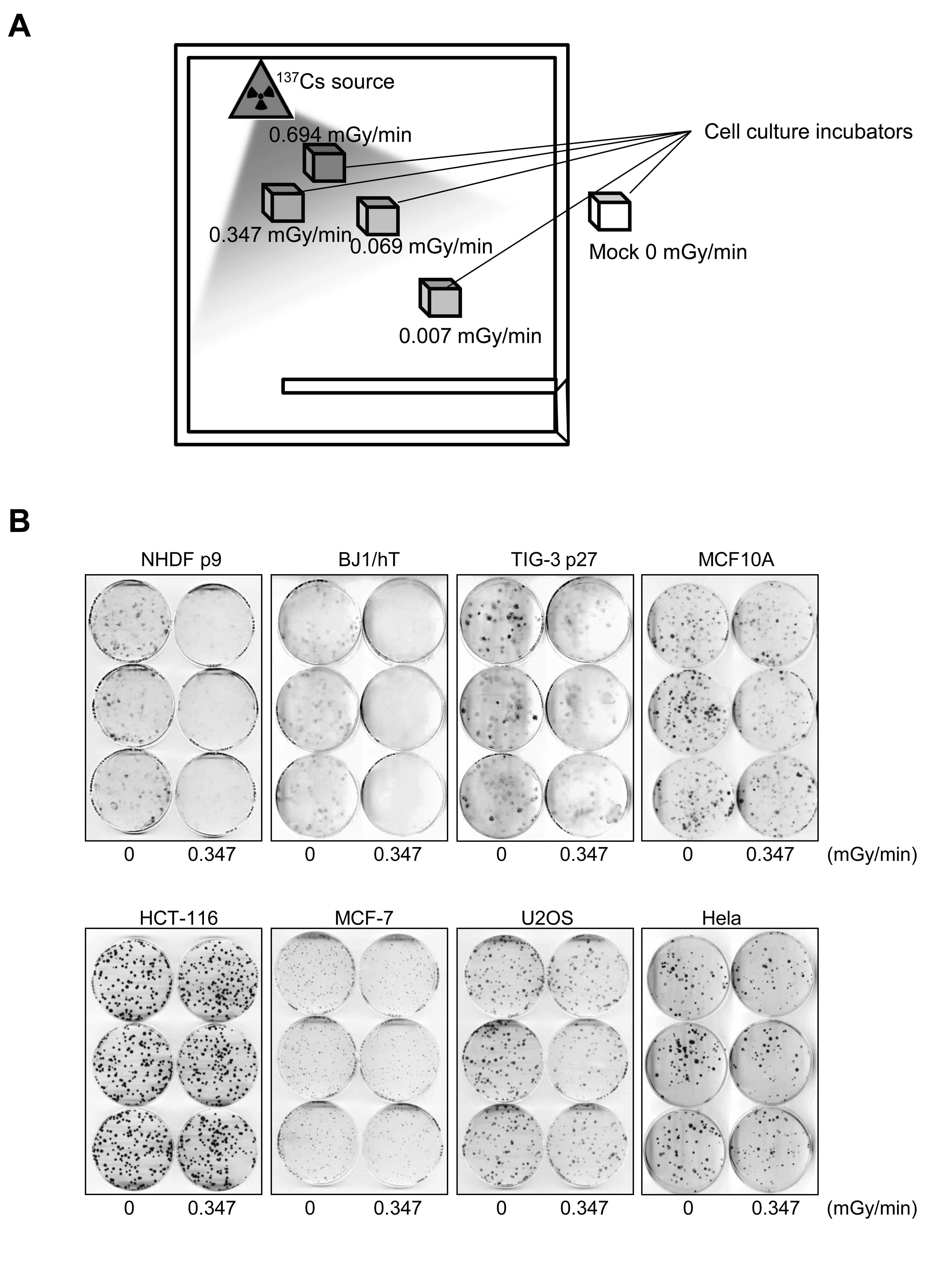

Supplement: Figure S1 — Different cell types exhibit different radiation sensitivity when exposed to chronic γ-irradiation. (A) A diagram of the culture room containing a 137Cs radiation source. Cubes indicate incubator positions. The dose rate for each incubator is indicated. (B) A variety of human cell lines were cultured for 10 days under control conditions (0) or chronic γ-irradiation conditions (0.347 mGy/min). Representative images of crystal violet-stained culture plates are shown. (TIF) [file pone.0104279.s001.tif]

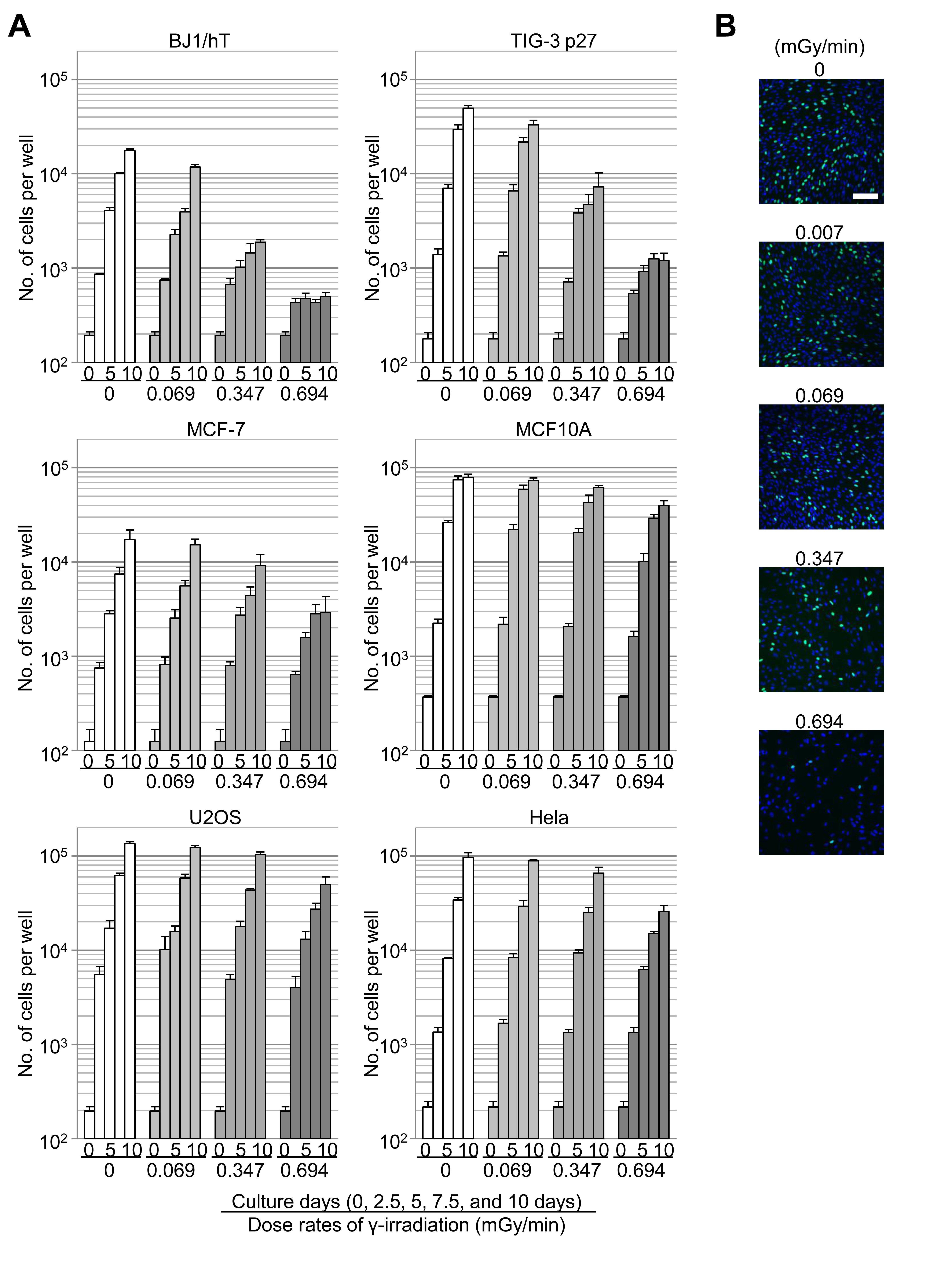

Supplement: Figure S2 — Chronic γ-irradiation specifically suppresses the proliferation of fibroblasts via a G1 arrest. (A) A number of human cell lines were cultured under chronic γ-irradiation conditions at indicated dose rates. After 0, 2.5, 5, 7.5, or 10 days the number of cells in each well was determined. Values represent the mean ± SD of six independent wells. (B) Representative images of TIG-3 p27 cells after 4 days of chronic γ-irradiation (dose rates are indicated). Hoechst 33258 staining of DNA (blue) and EdU-Alexa Fluor488 (green) are shown. Scale bar is 250 µm. (TIF) [file pone.0104279.s002.tif]

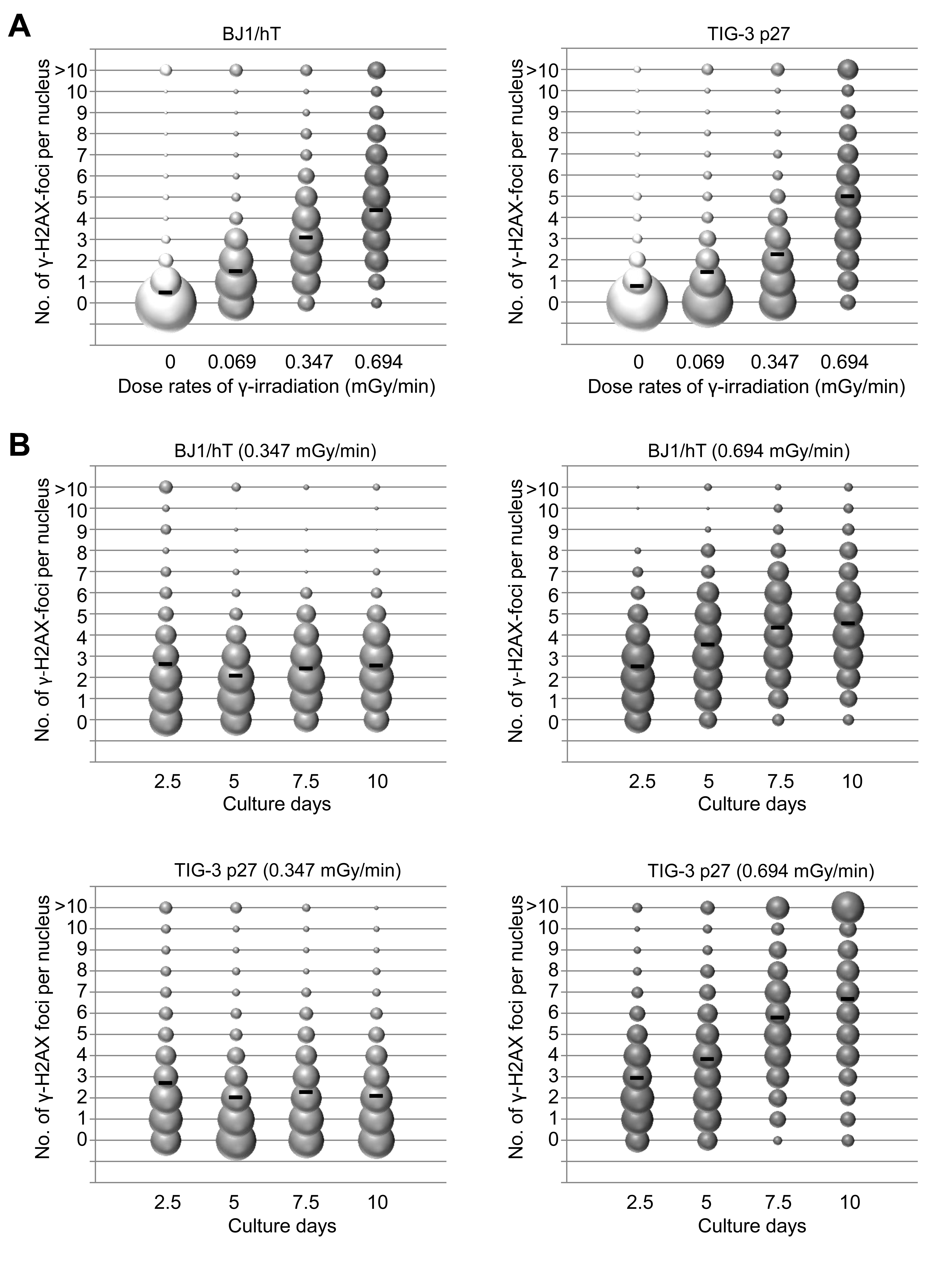

Supplement: Figure S3 — Human fibroblasts undergo senescence when accumulated DNA damage exceeds a threshold. (A) BJ1/hT (left) or TIG-3 p27 (right) cells were cultured for 4 days under chronic γ-irradiation conditions at indicated dose rates. The number of γ-H2AX-foci per cell was then determined. The size of the bubble is proportional to the number of cells with that number of γ-H2AX-foci. Black bars indicate the mean number of foci per cell. (B) The number of γ-H2AX-foci increased over time in response to chronic γ-irradiation conditions. BJ1/hT (upper) or TIG-3 p27 (lower) cells were cultured under chronic γ-irradiation conditions at indicated dose rates for 2.5, 5, 7.5, or 10 days. (TIF) [file pone.0104279.s003.tif]

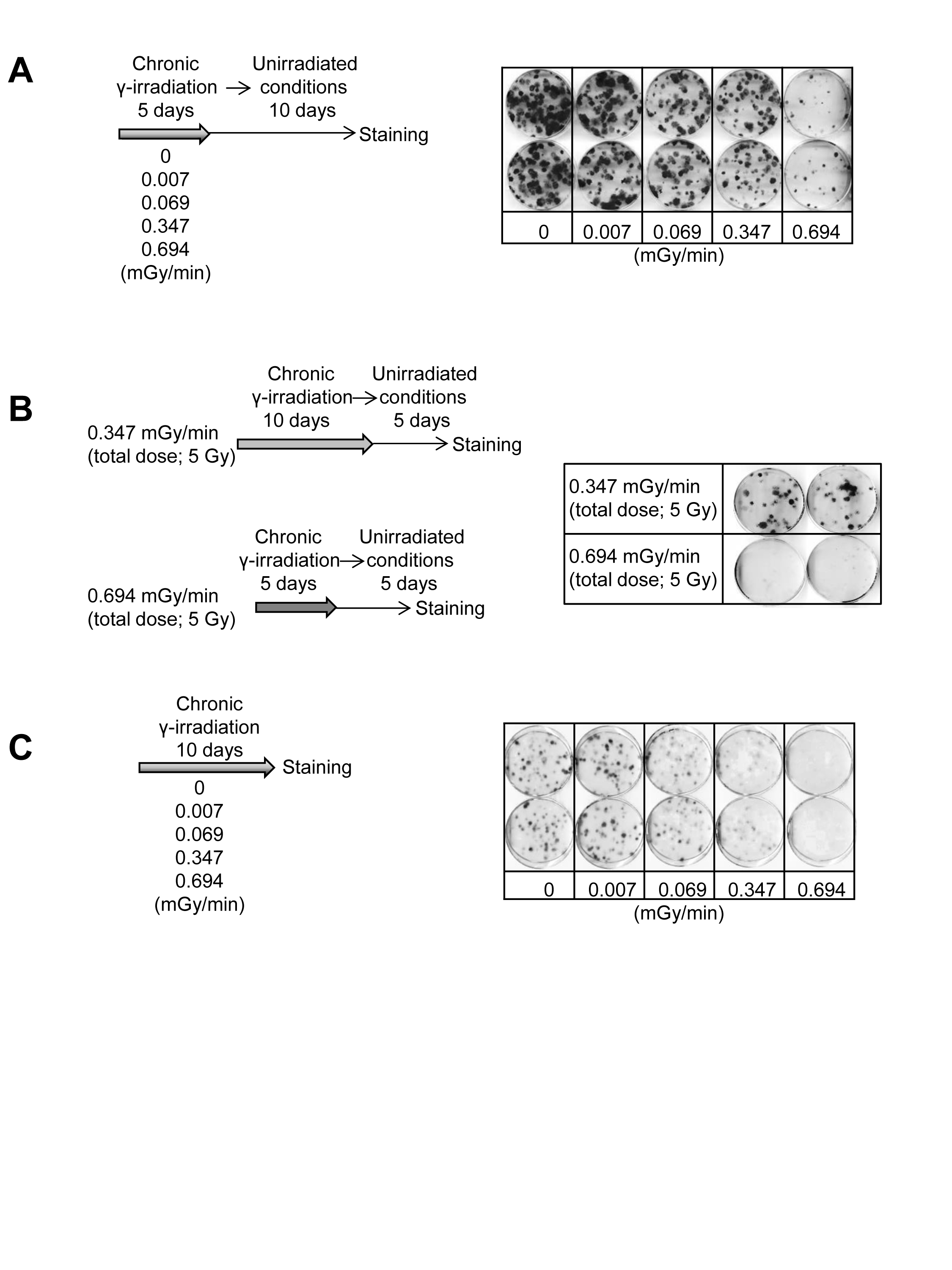

Supplement: Figure S4 — The chronic γ-irradiation dose rate affects cell-fate decisions in human fibroblasts. (A–C) Colony-forming ability of fibroblasts following chronic γ-irradiation. Experimental schemes are illustrated to the left. (A) TIG-3 cells (2×10^2^) were cultured at indicated dose rates for 5 days and then grown under unirradiated conditions for an additional 10 days. Culture plates were then stained using crystal violet. Representative images are shown to the right. (B) TIG-3 p27 cells (2×10^2^) were cultured for 10 days at 0.347 mGy/min or 5 days at 0.694 mGy/min (total dose of 5 Gy). Culture dishes were then incubated under unirradiated conditions for an additional 5 days and stained using crystal violet. Representative images of stained culture dishes are shown on the right. (C) TIG-3 p27 cells (2×10^2^) were cultured at indicated dose rates for 10 days. Representative images of crystal violet-stained culture dishes are shown to the right. (TIF) [file pone.0104279.s004.tif]

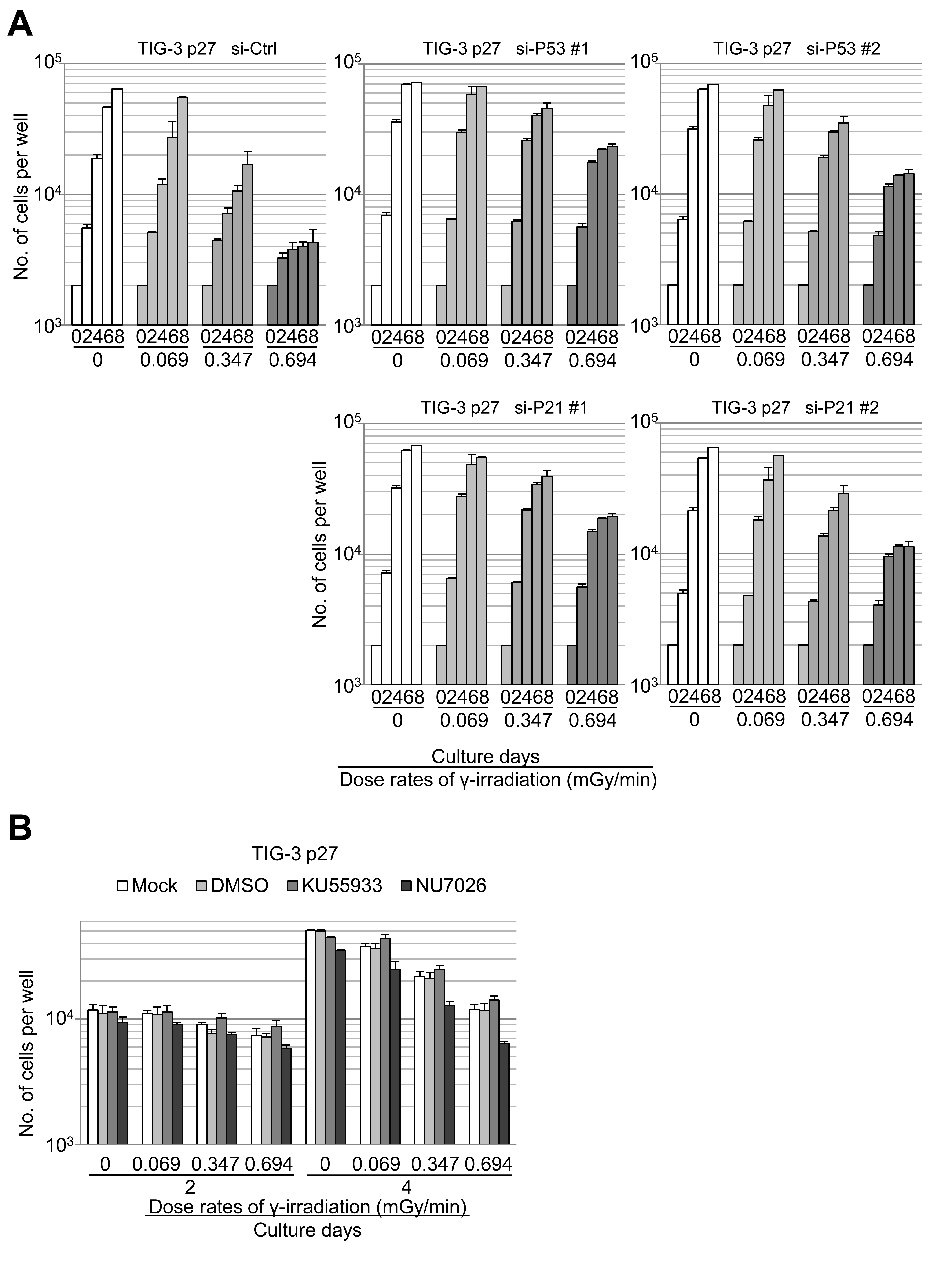

Supplement: Figure S6 — Inhibition of the ATM/TP53/p21 pathway attenuates chronic γ-irradiation induced growth inhibition. (A) TIG-3 p27 cells (2×10^3^) were transfected with indicated siRNAs and cultured under chronic γ-irradiation conditions at indicated dose rates. The number of cells per well was determined at indicated time points. Values represent the mean ± SD of three independent wells. (B) Inhibition of ATM kinase activity, but not DNA-PKcs activity, attenuates the growth inhibitory effect of chronic γ-irradiation. TIG-3 p27 cells were cultured for 2 or 4 days under chronic γ-irradiation conditions at indicated dose rates in the presence or absence of the ATM inhibitor KU55933 (10 µM) or the DNA-PKcs inhibitor NU7026 (10 µM). The number of Hoechst-stained nuclei was determined for each well. Values represent the mean ± SD of three independent wells. (TIF) [file pone.0104279.s006.tif]

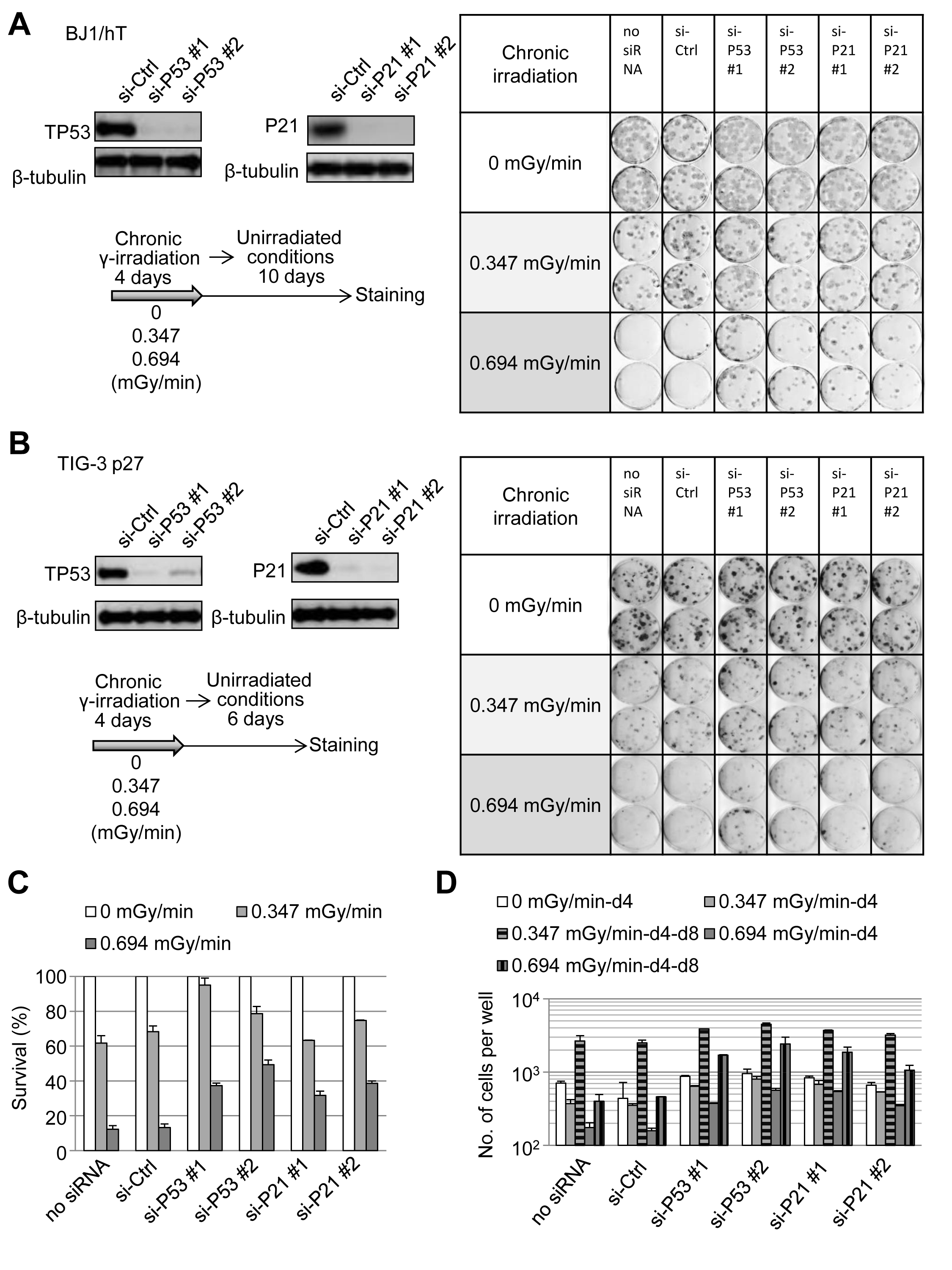

Supplement: Figure S7 — Knock down of TP53 or p21 attenuates chronic γ-irradiation-induced senescence. (A) Western blot analysis of BJ1/hT cells transfected with control siRNA (si-Ctrl) or siRNA specific for TP53 (si-P53 #1 or #2), or P21 (si-P21 #1 or #2) (upper left). β-tubulin served as the loading control. Cells transfected with indicated siRNA were cultured under chronic γ-irradiation conditions at indicated dose rates for 4 days, and then incubated an additional 10 days under non-irradiated conditions (experimental scheme is illustrated lower left). Representative images of crystal violet-stained colonies are shown (right). (B) TIG-3 p27 cells were analyzed as in (A), except that cells were cultured for 6 days following γ-irradiation. (C–D) TIG-3 p27 cells transfected with indicated siRNAs were exposed to γ-irradiation at indicated dose rates. The ability of these cells to form colonies (C) or to proliferate (D) was subsequently assessed as shown in Figure 5 (B–C). (TIF) [file pone.0104279.s007.tif]
